# Comparison of catheter-over-needle and catheter-through-needle methods in ultrasound-guided continuous femoral nerve block: A prospective, randomized controlled trial

**DOI:** 10.1097/MD.0000000000026519

**Published:** 2021-07-02

**Authors:** Hee Young Kim, Ji-Soo Ahn, Seyeon Park, Eun-Ji Choi, Hyun-Su Ri, Ji-Uk Yoon, Gyeong-Jo Byeon

**Affiliations:** a Department of Anesthesia and Pain Medicine, Pusan National University Yangsan Hospital, Pusan National University School of Medicine, Yangsan, Gyeongnam, Republic of Korea; b Research Institute for Convergence of Biomedical Science and Technology, Pusan National University Yangsan Hospital, Yangsan, Gyeongnam, Republic of Korea.

**Keywords:** catheter-over-needle, femoral nerve, leakage, nerve block, ultrasonography

## Abstract

**Background:**

The catheter-through-needle (CTN) method involves the insertion of a catheter with an outer diameter smaller than the initial puncture hole. We investigated whether the catheter-over-needle (CON) method is more effective than the CTN method in local anesthetic leakage at the catheter insertion site and catheter dislodgement, and how it affects postoperative pain management.

**Methods:**

Seventy patients scheduled to undergo continuous femoral nerve block for pain control following total knee arthroplasty were enrolled and randomized to receive a perineural catheterization with either the CTN method (group CTN) or CON method (group CON). After ultrasound-guided catheterization, the transparent securement dressing was attached. The study compared the CON and CTN methods in terms of leakage at the catheter insertion site, catheter dislodgement, and postoperative analgesic efficacy for 48 hours postoperatively.

**Results:**

Leakage at the catheter insertion site was significantly lower in the group CON (*P* < .05), while catheter dislodgement was not significantly different between the groups. The other adverse events were not different between the groups. The procedure time was significantly shorter in group CON (*P* < .05). No significant intergroup differences were observed 48 hours postoperatively in the visual analog scales, the number of patients requiring additional analgesics, and the number of times a bolus dose was injected with an injection pump.

**Conclusion:**

The CON method was able to shorten the procedure time while reducing the incidence of leakage at the catheter insertion site than the CTN method, and showed similar effects in postoperative pain management.

## 1 Introduction

Postoperative pain management is one of the key components in enhanced recovery after surgery for total knee arthroplasty (TKA).^[1]^ Patient-controlled analgesia using ultrasound-guided continuous femoral nerve block is known to reduce the duration of hospitalization and rehabilitation treatment by enabling early gait and joint movement by relieving severe pain immediately after surgery in patients undergoing TKA.^[2]^

Serious complications associated with continuous peripheral nerve blocks are generally known to be rare. However, common complications include local anesthetic leakage and catheter dislodgement. The rates of catheter dislodgement are reported in the literature as 6% to 15%.^[3,4]^ Leakage at the catheter insertion site not only reduces the volume of local anesthetic adjacent to the nerve, potentially causing block failure, but also induces disruption of the securement dressing, causing catheter dislodgement and potentially increasing infectious complications.^[5]^

The conventional catheter-through-needle (CTN) method involves the insertion of a catheter into the needle and placing the catheter around the femoral nerve. The outer diameter of the catheter is smaller than that of the initial needle-punctured hole, and there is a possibility of local anesthetic leakage at the catheter insertion site and catheter dislodgement. To overcome these problems, a catheter-over-needle (CON) method was devised. This method inserts a needle over the catheter, places it around the nerve, and removes only the needle. The catheter fits tightly in the puncture hole, reducing the incidence of local anesthetic leakage and catheter dislodgement.^[3,6]^

Previous studies on local anesthetic leakage and catheter dislodgement of the CON method have different results in several articles, and there is limited research on how the CON method is better in postoperative pain management than the conventional CTN method. This study aimed to investigate whether the CON method is more effective than the conventional CTN in local anesthetic leakage and catheter dislodgement and how it affects postoperative pain management and reduction of other adverse events. We hypothesized that the CON method would show less local anesthetic leakage and catheter dislodgement than the conventional CTN method, and would be better for postoperative pain management.

## 2 Methods

### 2.1 Patient enrollment

With the approval of the Institutional Review Board of the authors’ Hospital (ID 05-2018-130), the trial was registered with Clinical Research Information Service (registered number: KCT0003509). After obtaining written informed consent, we enrolled 70 American Society of Anesthesiologists physical status I–III patients undergoing TKA. Patients with poor coordination, pregnant women, blood coagulation disorders, neurologic defects at the site, and allergic reactions to ropivacaine in previous surgeries were excluded.

### 2.2 Randomization

At the preanesthetic visit, all subjects were fully described on the randomization protocol, pain assessment using the visual analog scale (VAS), and how to use a portable electronic injection pump, and agreed to participate in the study. Random assignment to 2 groups of patients used a list of random numbers generated using Excel (Microsoft Corporation, Redmond, WA, USA). Patients underwent TKA with ultrasound-guided continuous femoral nerve blocking using either the CTN method (group CTN) or the CON method (group CON). The study was a double-blind, randomized controlled study. For randomization, the investigator who performed the procedure could not measure the outcome after surgery, and the outcome investigator was blinded to the procedure.

### 2.3 Catheter insertion procedure

Before induction of general anesthesia, all patients underwent the ultrasound-guided continuous femoral catheter insertion in the supine position with the leg slightly externally rotated. The femoral nerve was detected using a 5.0 to 13.0 MHz linear probe (LOGIQ e; GE Healthcare, Princeton, NJ, USA). After disinfecting the skin around the inguinal area with chlorhexidine-alcohol, group CTN (n = 35) had the catheter mounted under the femoral nerve using the CTN method, and group CON (n = 35) had it mounted using the CON method.

In group CTN, after infiltration of the needle insertion site with 3 to 4 mL of 2% lidocaine, a 10-cm, 18-gauge Tuohy needle (NRFit PlexoLong Nanoline Kit; Pajunk GmbH, Geisingen, Germany) was inserted and placed along the lower lateral part of the femoral nerve under ultrasound guidance. Electrocardiogram pads were placed 0 to 1 cm medial to the distal quadriceps tendon and attached to a nerve stimulator (Medipia ES400; Life-Tech, Stafford, TX, USA). Initial output of 1 mA, 2 Hz, and 0.2 ms was applied as the block needle was advanced along the lower part of the femoral nerve until quadriceps femoris muscle contractions were elicited, during which the nerve stimulator was turned off. A 20-gauge stimulating catheter (NRFit PlexoLong Nanoline Kit; Pajunk GmbH, Geisingen, Germany) was inserted through the needle. The catheter tip was localized at the lower mid-point of the femoral nerve, adjusted using the ultrasound image, and injected 1 to 2 mL of normal saline. If the tip of the catheter could not be seen, the process of catheter insertion and localization was repeated. After catheter placement, 10 mL of 0.2% ropivacaine was injected under ultrasound guidance to confirm that the local anesthetic diffused well around the nerves. To secure the catheter, the catheter insertion site was attached with a chlorhexidine gluconate transparent securement dressing (Tegaderm CHG; 3M Corporation, St. Paul, MN, USA). Thereafter, an additional 10 mL of 0.2% ropivacaine was injected through the catheter to examine whether local anesthetic leakage had occurred.

In group CON, in the same manner as in group CTN, a 5-cm, 18-gauge cannula with an indwelling 21-gauge needle (E-cath Plus; Pajunk GmbH, Geisingen, Germany) was inserted and placed along the lower lateral part of the femoral nerve under ultrasound guidance. Electrocardiogram pads were placed 0 to 1 cm medial to the distal quadriceps tendon and attached to a nerve stimulator, applied in the same way. A 21-gauge E-catheter with integrated tubing (E-cath Plus; Pajunk GmbH, Geisingen, Germany) was inserted through the indwelling 18-gauge cannula. The E-catheter tip was localized at the lower mid-point of the femoral nerve, adjusted using the ultrasound image, and injected 1 to 2 mL of normal saline. After catheter placement, 10 mL of 0.2% ropivacaine was injected under ultrasound guidance to confirm that the local anesthetic diffused well around the nerves. The catheter insertion site was attached with a chlorhexidine gluconate transparent securement dressing and then injected with an additional 10 mL of 0.2% ropivacaine to check for local anesthetic leakage.

### 2.4 Perioperative management

General anesthesia was performed using 6 vol% desflurane. At the end of the surgery, 225 mL of 0.2% ropivacaine was infused through the indwelling catheter via a portable electronic injection pump (Accumate 1100; Woo Young Medical Co., Ltd., Chung-Buk, Korea) for the first 48 hours after surgery in both groups. Both groups received a periodically at 4-hour interval dose at 5 mL of 0.2% ropivacaine, and a patient-requiring bolus dose at 5 mL with a lockout time of 30 min through the catheter using a portable electronic injection pump. All surgical procedures were performed by the same orthopedic surgeon. Before subcutaneous closure, the intra-articular injection was performed with 30 mL of 0.2% ropivacaine by the surgeon.

### 2.5 Outcome measurements

The primary outcome was leakage at the catheter insertion site under the transparent securement dressing, detected by visual inspection. Other catheter-related adverse events such as dislodgement, kinking, knotting, and cutting were monitored and recorded for 48 hours postoperatively. The procedure time was defined as the time from infiltration of a local anesthetic to the time that a chlorhexidine gluconate securement dressing was applied over the catheter insertion site.

An investigator who was blinded to the group assignments was assigned to assess postoperative pain quality using a VAS, as well as the incidence of patients requiring additional analgesics, totally consumed doses of local anesthetics, adverse events related to local anesthetics, and patient satisfaction regarding postoperative pain management. The VAS was recorded immediately after admission in the postanesthetic care unit, and at 1, 4, 12, 24, 36, and 48 hours postoperatively. When the VAS score was >60 and the patient wanted analgesics during the postoperative period, morphine 0.05 mg/kg was injected. Additional analgesic requirements within 48 hours after surgery were documented as the incidences of patients requiring additional analgesics by the investigator. Adverse events related to local anesthesia including nausea, vomiting, dizziness, hypotension, urinary retention, and paresthesia were noted. Patient satisfaction regarding postoperative pain management was assessed on a 5-point Likert scale as follows:^[7]^ 5 = very satisfied, 4 = satisfied, 3 = neutral, 2 = dissatisfied, and 1 = very dissatisfied.

### 2.6 Sample size estimation

The primary outcome was the rates of leakage at the catheter that was placed for the ultrasound-guided, continuous femoral nerve block. In previous studies, the leakage rates in the CTN and CON methods were 55% and 0%, respectively.^[8]^ In this study, assuming that the difference in the leakage rate was approximately 30% between the 2 methods, the sample sizes were measured to be 32 patients with type I (α) and type II (β) errors of 0.05 and 0.2, respectively. Taking into account the 10% dropout rate, the sample size was 35 patients in each group. Altogether 70 patients were recruited in this study.

### 2.7 Statistical analysis

Statistical analysis was performed using IBM SPSS Statistics for Windows, version 26.0 (IBM Corp., Armonk, NY, USA). For the demographic data, a Student *t* test was used for the numerical data, and the chi-squared test was used for the categorical data. A *t* test was used to compare VAS and total consumed local anesthetics. The incidences of adverse events, patient satisfaction with postoperative pain management, times of bolus injection using local anesthetic delivery injection pump, and rescue opioid administration were compared using the chi-squared test or Fisher exact test. *P* < .05 was considered statistically significant.

## 3 Results

**Figure 1:**
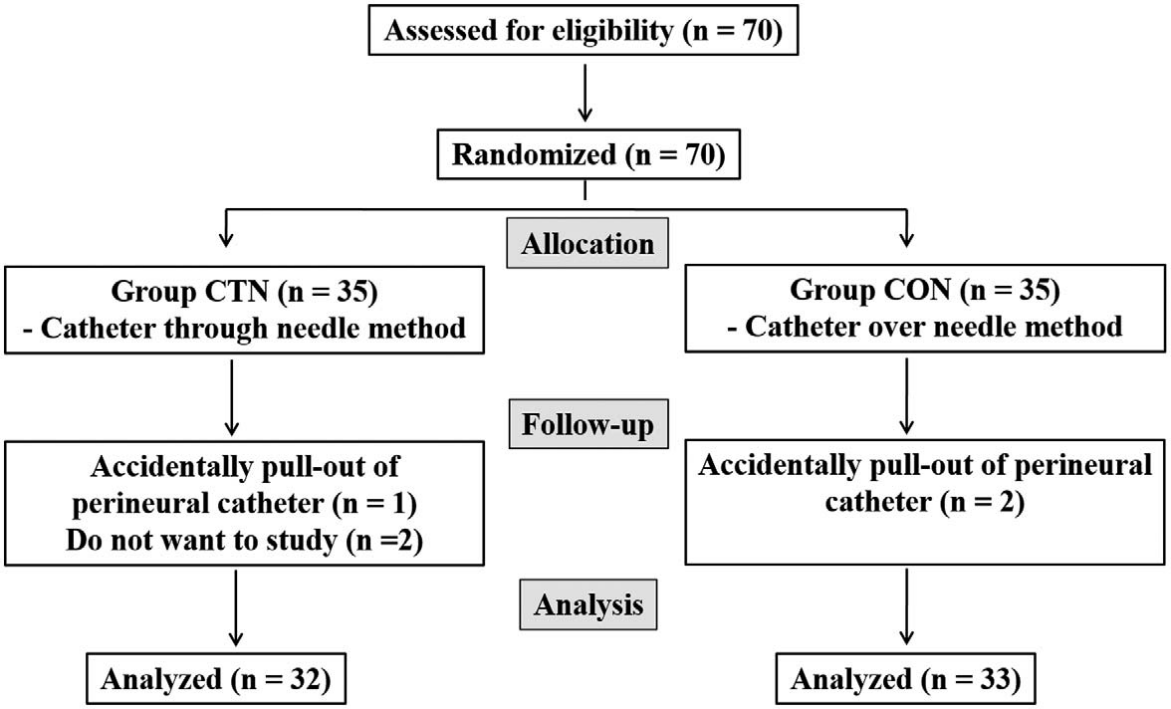
Patient enrollment and a study flowchart.

Seventy patients were enrolled in this study. One patient in group CTN and 2 patients in group CON dislodged the perineural catheter within the first 24 hours after the surgery. They were excluded from the study and received postoperative pain management through intravenous patient-controlled analgesia using nonsteroidal anti-inflammatory and opioids for rescue analgesia. Two patients in group CTN did not want to continue this study after surgery; the remaining 65 patients completed the study (Fig. 1). Regarding demographic data, no differences were observed between the 2 groups in American Society of Anesthesiologists physical status, sex, age, height, weight, and anesthesia time. The procedure time was statistically shorter in group CON (*P* < .05, Table 1).

**Table 1:** Demographic data.

Characteristic	Group CTN (n = 32)	Group CON (n = 33)	*P* value
ASA physical status (I/II/III)	7/21/4	6/25/2	.584
Sex (M/F)	3/29	3/30	.968
Age (years)	68.6 ± 7.0	69.1 ± 6.0	.731
Height (cm)	153.5 ± 5.9	153.3 ± 6.8	.411
Weight (kg)	63.1 ± 8.8	61.4 ± 8.8	.708
Procedure time (min)	9.8 ± 2.2	6.8 ± 1.0^∗^	<.001
Anesthesia time (hour)	3.3 ± 0.3	3.2 ± 0.3	.574

All measured values are presented as mean ± standard deviation or number of patients. ASA = American Society of Anesthesiologists, CON = catheter-over-needle, CTN = catheter-through-needle.

∗ *P* < .05 compared with group CTN.

Leakage at the catheter insertion site occurred in 11 patients in group CTN and 2 patients in group CON, respectively. Leakage at the catheter insertion site was significantly lower in the group CON (*P* < .05). As mentioned above, catheter dislodgement occurred in 1 patient in group CTN and 2 patients in group CON, respectively. In 1 patient in group CTN, an “occlusion” alarm occurred in the portable electronic infusion pump, which was caused by kinking a catheter attached across the inguinal fold when the patient sat down. This was resolved by removing the fixation tape, straightening the kinking portion, and fixing it with new tape. There were no other adverse events related to the perineural catheter and no statistical significance between the 2 groups. Adverse events related to local anesthetics were not different between the groups (Table 2).

**Table 2:** Incidence of adverse events.

Adverse events	Group CTN (n = 32)	Group CON (n = 33)	*P* value
Related with the perineural catheter			
Leakage	11 (34.4)	2 (6.1) ^∗^	.004
Dislodgement	1 (3.1)	2 (6.1)	1.000
Kinking	1 (3.1)	0 (0.0)	.492
Knotting	0 (0.0)	0 (0.0)	1.000
Cutting	0 (0.0)	0 (0.0)	1.000
Related with the local anesthetics			
Nausea	4 (12.5)	5 (15.2)	1.000
Vomiting	0 (0.0)	0 (0.0)	1.000
Dizziness	6 (18.8)	4 (12.1)	.511
Hypotension	0 (0.0)	0 (0.0)	1.000
Urinary retention	4 (12.5)	3 (9.1)	.708
Paresthesia	4 (12.5)	2 (6.1)	.427

Values are presented as the number of patients (%). CON = catheter-over-needle, CTN = catheter-through-needle.

∗ *P* < .05 compared with group CTN.

**Figure 2:**
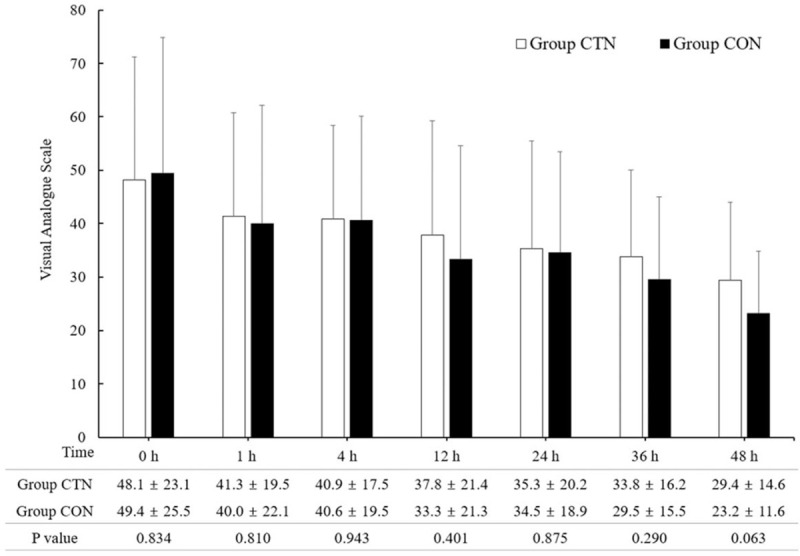
There were no significant intergroup differences were observed in the VAS scores immediately after admission to the PACU, and at 1, 4, 12, 24, 36, and 48 hours after the surgery. Group CTN = catheter-through-needle method, Group CON = catheter-over-needle method, 0 h: immediately after admission to the PACU. PACU = postanesthetic care unit, VAS = visual analog scale.

No significant intergroup differences were observed in the VAS immediately after admission to the postanesthetic care unit, and at 1, 4, 12, 24, 36, and 48 hours after the surgery (Fig. 2). The incidence of patients requiring additional analgesics within 48 hours after surgery was not significantly different between the 2 groups (Table 3).

**Table 3:** Incidences of patients requiring additional analgesic within 48 hours after surgery.

Incidence	Group CTN (n = 32)	Group CON (n = 33)	*P* value
			.391
0	12 (37.5)	10 (30.3)	
1	4 (12.5)	9 (27.3)	
2	5 (15.6)	4 (12.1)	
3	5 (15.6)	6 (18.2)	
4	3 (9.4)	3 (9.1)	
5	3 (9.4)	0 (0.0)	
6	0 (0.0)	0 (0.0)	
7	0 (0.0)	1 (3.0)	

Values are presented as the number of patients (%). CON = catheter-over-needle, CTN = catheter-through-needle.

The times a bolus dose was injected within 48 hours after surgery were not significantly different. There were also no significant differences between the groups in the total consumed dose of local anesthetics from the portable electronic injection system (Table 4). Patient satisfaction regarding postoperative pain management was not significantly different between the 2 groups.

**Table 4:** The bolus dose was injected within 48 hours after surgery and the total consumed dose of local anesthetics from the portable electronic injection system are shown.

Characteristic	Group CTN (n = 32)	Group CON (n = 33)	*P* value
The times a bolus dose has been injected (times)	8.0 ± 4.1	8.5 ± 5.4	.668
Total consumed dose of local anesthetic			
0 h (mL)	5.0 ± 0.0	5.0 ± 0.1	.677
1 h (mL)	8.0 ± 2.8	8.0 ± 2.5	.912
4 h (mL)	13.7 ± 6.6	14.9 ± 7.0	.477
12 h (mL)	52.5 ± 20.5	49.9 ± 22.7	.635
24 h (mL)	94.7 ± 25.2	87.9 ± 33.1	.358
36 h (mL)	133.8 ± 27.3	134.9 ± 33.2	.884
48 h (mL)	175.8 ± 34.6	176.9 ± 34.9	.898

All measured values are presented as mean ± standard deviation. 0 h: immediately after admission to the PACU, CON = catheter-over-needle, CTN = catheter-through-needle, PACU = postanesthetic care unit.

## 4 Discussion

This randomized, comparative study was undertaken to investigate whether the CON method is more effective than the conventional CTN method with regards to local anesthetic leakage and catheter dislodgement, and how it affects postoperative pain management and reduction in other adverse events. The results of this study showed the leakage at the catheter insertion site was significantly lower in the CON method, however, no difference in the catheter dislodgement between the CTN and CON methods. And there were no differences in postoperative pain management and other various adverse events between the 2 methods.

Complications such as local anesthetic leakage and catheter dislodgement are common in continuous peripheral nerve blocks. Incorrect positioning of catheters occurs in up to 40%, leading to a disruption of the dressing which can lead to catheter dislodgement and potentially increase infective complications. Leakage reduces the volume of local anesthetic adjacent to the nerve, potentially causing block failure. Inadvertent catheter dislodgement is another common complication of continuous catheter techniques.^[9,10]^

In the study, the CON method showed 6.1% leakage and 6.1% dislodgement, whereas the CTN method showed 34.4% leakage and 3.1% dislodgement. As shown in the results, it can be seen that the CON method had little leakage. The catheter in the CON method has a diameter larger than that of the puncture needle. This may increase resistive forces when traction is unintentionally applied to the catheter, decreasing the chance of dislodgement.^[6]^ In addition, we used a transparent securement dressing attached to sticky gel-type chlorhexidine gluconate at the catheter insertion site that reportedly prevents such leakage during continuous infusion. These 2 steps prevented leakage at the catheter insertion site and catheter dislodgement in the CON method.

In a recent study, leakage at the catheter insertion site was observed in 55% of patients using the CTN method, whereas there was no leakage using the CON method.^[8]^ Although it was dependent on different operators and different insertion sites, leakage at the catheter insertion site has been reported to occur in 3% to 30% of perineural catheters using the CTN method.^[11]^ As reported in several articles, the CTN method has the problem of leakage at the catheter insertion site, so suturing the catheter has been mainly used to resolve this problem. In addition to suturing the catheter, several methods have been used to overcome the adverse events associated with the perineural catheter, including the subcutaneous tunneling,^[12]^ application of adhesive glue,^[13,14]^ and addition of adhesive anchoring devices such as wound closure strip (Steri-Strips; 3M Corporation, St. Paul, MN, USA), catheter-hub connections devices (eg, StatLock).^[15,16]^ However, if the strength of the suture is too strong, there is a possibility that the catheter is clogged, and subcutaneous tunneling is a risk due to additional procedures.

The design of a conventional CTN assembly in which a flexible smaller diameter catheter is passed through a larger diameter needle may be prone to leakage and dislodgement at the catheter insertion site. On the other hand, the CON method formed a tighter seal at the needle insertion site. The diameter of the catheters used in the CON method is larger than that of the needle, sealing the catheter in place, and reducing the risk of leakage and dislodgement at the insertion site. According to the study results, the catheter used in the CON method had a holding force that was 6 times greater than the CTN method.^[6]^ A greater holding force means that the catheters used in the CON method will not fall out as frequently, especially if the catheters are fixed only with dressing.

Another advantage is that the CON design allows the clinician to pull out the needle while simultaneously holding the catheter in place. The fact that the skin at the insertion site secures the catheter securely also allows the clinician to pull the needle out with 1 hand so that the catheter does not move back and forth to the point where it enters the skin. There was also an advantage in reducing the procedure time, since no catheter fixation was required, and procedure time was significantly lower when employing the CON method. In our study, the procedure time was shorter in the CON method. In addition to the above advantage, it is considered that the CON method was able to reduce the procedure time because it did not need to go through an ultrasound verification process to confirm that it was properly mounted under the femoral nerve. On the other hand, the catheter was relatively thick and less flexible. If the skin area was not flat, the catheter was somewhat floating away from the skin. In these cases, there seems to be a risk of dislodgement. These characteristics of the CON catheter are considered to be suitable for continuous femoral nerve block.^[17]^

The CON method was used to place the catheter tip under the femoral nerve. Such placement helped prevent perineural catheter tip dislocation and might have reduced the incidence of unintended nerve blocking caused by inappropriate catheter tip position. Thus, the incidence of adverse events can be expected to be lower using the CON method over the CTN method. However, there were no significant differences in the incidence of adverse events related to local analgesics.

Meanwhile, there were no differences between the 2 methods in VAS, the incidence of patients requiring additional analgesics, times of bolus dose injection, and total consumed dose of local anesthetics from the portable electronic injection system. The leakage at the catheter insertion site was relatively lower, and due to the larger catheter diameter, local anesthetics were thought to spread better and provide better pain control, but the actual results did not confirm this.

Our study has several limitations. First, the needles used when using the CTN method and the needles used when using the CON method were different. A 10-cm, 18-gauge needle with a Tuohy-type tip was used in the CTN method, and a 5-cm, 21-gauge needle with a facet grinding-type tip was used in the CON method. The 2 needles differed by 5 cm in length, and the shapes of the needle tips were different. These differences might also have affected the procedure time. Second, although comparing the catheter-related adverse events requires that both methods proceed under the same conditions, it is difficult to set the same conditions. In our study, the CTN method resulted in leakage at the catheter insertion site in 34.4% of patients. Local anesthetics leaked out at the catheter insertion site so that the dressing area was wet, and it was necessary to attach the transparent securement dressing again. Third, because of the different shapes of the 2 types of catheters, it was impossible to blind the catheter-related review during or immediately after the procedure. Instead, several assessments were performed in a blind manner, covering the area of the procedure with clothes such that neither patients nor researchers could know which catheter was used.

In conclusion, the CON method was able to shorten the procedure time while reducing the incidence of leakage at the catheter insertion site to the CTN method and showed similar effects in postoperative pain management.

## Author contributions

**Conceptualization:** Hee Young Kim, Gyeong-Jo Byeon.

**Data curation:** Seyeon Park, Eun-Ji Choi, Hyun-Su Ri.

**Formal analysis:** Eun-Ji Choi, Hyun-Su Ri, Ji-Uk Yoon.

**Investigation:** Ji-Soo Ahn, Seyeon Park.

**Methodology:** Hee Young Kim, Gyeong-Jo Byeon.

**Supervision:** Ji-Uk Yoon.

**Writing – original draft:** Hee Young Kim, Gyeong-Jo Byeon.

**Writing – review & editing:** Gyeong-Jo Byeon.
